# Effect of Asphalt Content on Low-Field Nuclear Magnetic Resonance Spectrum and Aging Evaluation of Asphalt Mixtures

**DOI:** 10.3390/ma18092004

**Published:** 2025-04-28

**Authors:** Chenyue Mei, Wei Wang, Junan Shen, Jinkun Sun, Yilin Xu, Jia Che

**Affiliations:** 1 Suzhou Highway Development Center, Suzhou 215007, China; 13451606498@163.com; 2 School of Civil and Architectural Engineering, Panzhihua University, Panzhihua 617000, China; wwangcqjtu@outlook.com (W.W.); paxf66290838@163.com (J.S.); 3 Department of Civil Engineering and Construction, Georgia Southern University, Statesboro, GA 30458, USA; 4 Jiangsu Technology Industrialization and Research Center of Ecological Road Engineering, Suzhou University of Science and Technology, Suzhou 215011, China; lcc13115019691@163.com (Y.X.); cj15050472577@outlook.com (J.C.)

**Keywords:** low-field nuclear magnetic resonance, asphalt mixtures, asphalt content, aging, principal component analysis

## Abstract

The aging of asphalt mixtures has a significant impact on the service life of asphalt pavements. Currently, the commonly employed method for assessing aging involves the extraction of asphalt from asphalt mixtures using the Abson method. However, this method is known to be detrimental to the extracted asphalt samples, time-consuming, and environmentally unfriendly. This study explored a novel non-destructive method for assessing asphalt aging, known as low-field nuclear magnetic resonance (LF-NMR). It primarily investigated the influence of asphalt content in asphalt mixtures on the patterns of LF-NMR spectra. Specifically, it examined the effect of asphalt content on LF-NMR spectra in asphalt mixtures with varying particle sizes and aging levels at the same detection temperature. Additionally, machine learning was used to establish predictive models linking NMR spectral features to asphalt mixture aging levels, enhancing interpretation accuracy. The research results revealed the following: (1) Spectral parameters such as peak height, normalized peak area, and normalized total peak area had a significant impact on the first principal component of LF-NMR spectra. (2) Asphalt content in the mixture increased as particle size decreased, leading to corresponding changes in the LF-NMR spectra. (3) There was a strong correlation between the aging degree of asphalt and asphalt mixtures and the normalized total peak area of their LF-NMR spectra. The study provides a non-destructive method to assess asphalt mixture aging, enabling timely maintenance decisions and improving pavement durability.

## 1. Introduction

Under the coupling effect of temperature, oxygen in the air, ultraviolet light, rainwater, and other natural factors, asphalt mixtures undergo aging. Aging leads to increased brittleness and reduced durability, which significantly diminishes the performance of asphalt pavements, causing issues such as cracking, potholes, and raveling, thereby shortening the service life of asphalt pavements [1,2]. Therefore, monitoring and evaluating the aging state of asphalt mixtures are crucial for the timely implementation of appropriate maintenance measures to improve pavement performance and extend its lifespan. Correctly assessing aging is of great importance for researching ways to enhance the aging resistance of asphalt mixtures. Currently, the aging state of asphalt is typically evaluated by extracting asphalt from the mixture using the Abson extraction method, followed by the assessment of the asphalt and its mixture [3,4]. However, this traditional extraction method uses chemical reagents (such as trichloroethylene) and involves a heating process that further ages the asphalt, compromising its original properties and failing to accurately reflect the aging state of the asphalt in the mixture. Moreover, the extraction process is complex and time-consuming, and some of the chemical solvents used are harmful to both laboratory personnel and the environment [5].

Nuclear magnetic resonance (NMR) spectroscopy detects the interaction between protons in atomic nuclei and an external magnetic field. In recent years, it has been used to analyze the chemical structure of asphalt and infer its aging mechanisms [6]. NMR’s key feature is its ability to qualitatively and quantitatively analyze the composition and structure of substances [7]. Low-field NMR (LF-NMR) measures spin–spin relaxation (transverse relaxation time, T2) in the sub-microscopic field (between molecules). This method offers the advantages of being non-destructive, low-cost, and efficient. Since its discovery in 1946, NMR has become a valuable tool in physics, chemistry, biology, and medicine [8]. Recently, LF-NMR has been used to assess the aging state of asphalt mixture samples as a non-destructive, in situ detection method that is gaining increasing attention [9,10,11,12,13,14]. During the aging process of asphalt mixtures, the volatile components of the asphalt decrease, and small molecules aggregate into larger molecules, increasing the viscosity of the asphalt [14]. Additionally, the oxidation of asphalt leads to the formation of carbonyl (C=O) and sulfoxide (S=O) groups, reducing the Relative Hydrogen Index (RHI) [12]. In the T2 spectrum of LF-NMR, the transverse relaxation time T2 is related to the viscosity of the measured fluid, while the amplitude is related to the RHI [12]. Therefore, the NMR spectrum of asphalt reflects its aging degree.

Menapace et al. [12] applied LF-NMR to study the base asphalt mixtures with different aging times and void ratios. The results showed that as asphalt aging progresses, the transverse relaxation time T2 shortens and the amplitude (RHI) decreases. They established a relationship between RHI and the viscosity of the asphalt binder: As viscosity increases, the volumetric relaxation rate also increases linearly, resulting in a decrease in the measured transverse relaxation time T2 (or RHI).

Menapace proposed that the aging of asphalt binders is related to the Relative Hydrogen Index (RHI) and presented an equation between the RHI and the amplitude of the NMR T2 mapping [11]:

RHI = AI _asphalt_/AI _water_ (1)

AI is the amplitude index with the following formula:

AI = Accumulated Amplitude/Mass (2)

The cumulative amplitude is the estimated spin-echo sequence amplitude at t = 0 ms, which is also the sum of all T2 components in the T2 distribution, providing a projection of T2 decay back to t = 0. RHI is proportional to the cumulative amplitude [13]. The spin echo decay (SED) curve is transformed into the T2 spectrum via the Fourier transform. According to the calculation formula for RHI and the principles of NMR, the RHI value and the estimated spin-echo sequence amplitude at t = 0 of the normalized T2 spectrum from LF-NMR are consistent, meaning the cumulative amplitude is the sum of all T2 components, i.e., the normalized peak total area [15,16]. As the degree of aging increases, the normalized peak total area decreases, resulting in a reduction in the cumulative amplitude and RHI.

In existing studies using low-field NMR to detect asphalt and its mixtures, the relaxation peaks of the T2 spectrum have been normalized. The formula for the normalized peak total area (NPA) is as follows [5,17]:

NPA = A/m (3)

A—total area of relaxation peak;
M—sample mass (g).

In this method, the NMR spectrum of asphalt mixtures primarily originates from the hydrogen signals in the asphalt, with the contribution of aggregate signals being negligible compared to that of the asphalt [5,17]. The role of asphalt content is therefore significant.

Despite growing interest in applying LF-NMR techniques to asphalt materials, limited attention has been paid to the influence of asphalt content on NMR spectral characteristics. Moreover, few studies have explored the integration of machine learning algorithms to interpret NMR data for asphalt aging evaluation. Addressing these gaps, this study proposes a novel, non-destructive approach that leverages LF-NMR combined with machine learning to assess aging behavior in asphalt mixtures, offering both scientific insight and practical value for long-term pavement performance monitoring. On this basis, the aim of this study is to investigate the impact of asphalt content on the NMR spectrum of mixtures. Specifically, it discusses the designed asphalt content in asphalt mixtures, the calculation of asphalt content for mixtures with different particle sizes, and the effect of measured asphalt content using the ignition method on the spectrum. The relationship between different aging degrees and asphalt content on the normalized peak total area is verified to evaluate asphalt aging.

## 2. Materials and Methods

### 2.1. Materials

The asphalt mixture used in this experiment is based on the Superpave 9.5 gradation, with an optimum asphalt content of 5.3% [18]. Basalt, sourced from Inner Mongolia Province, China, was selected as the aggregate, and Shuanglong 70# base asphalt, supplied by Suzhou Sanchuang Road Engineering Co., Ltd. in Suzhou, Jiangsu Province, China, was used as the asphalt binder. The basic properties are shown in Table 1. To investigate the effect of different aggregate sizes on the nuclear magnetic resonance (NMR) spectra due to varying asphalt content, a vibrating sieve shaker was used to classify the asphalt mixture into three particle size ranges: 0–3 mm, 3–5 mm, and 5–10 mm, corresponding to the gradation ranges of basalt aggregate used in the Superpave 9.5 mm nominal maximum aggregate size (NMAS) specification.

### 2.2. Methods

**Figure 1:**
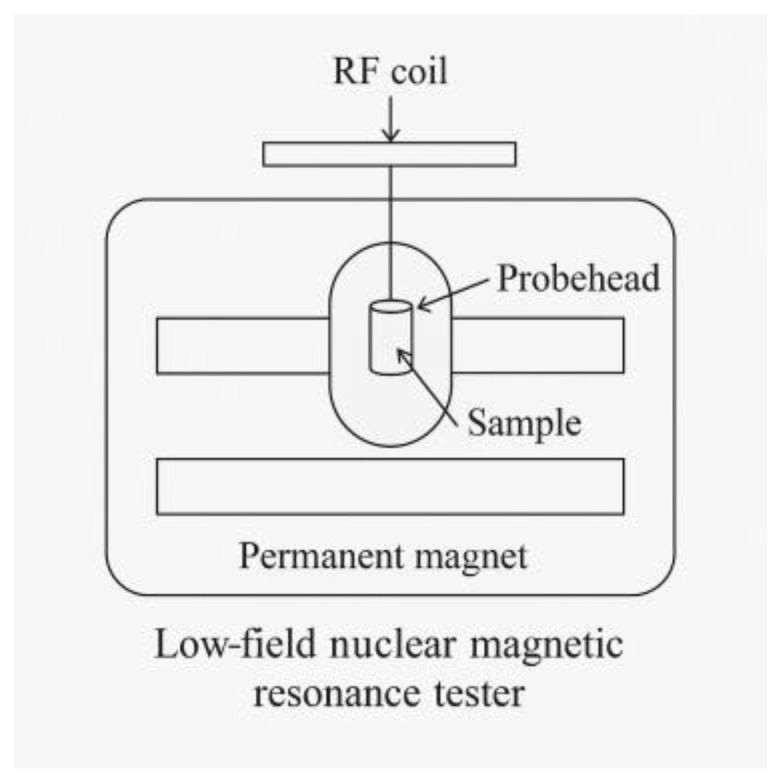
Schematic diagram of a low-field nuclear magnetic resonance tester.

The experiment was conducted using a low-field nuclear magnetic resonance (NMR) analyzer (Model PQ001-20-010V, Suzhou Niumai Analytical Instrument Co., Ltd., Suzhou, China), as shown in Figure 1. The CPMG pulse sequence was employed, with an echo interval of 0.150 ms, 3000 echoes, a primary frequency of 21 MHz, a receiver bandwidth of 200 kHz, a polarization time of 2500 ms, 32 accumulations, and a test temperature of 60 °C [19].

**Figure 2:**
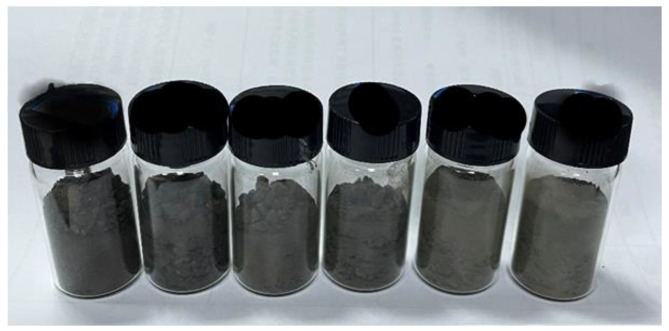
Samples with different particle sizes.

After sample preparation, asphalt mixtures with particle sizes of 0–3 mm, 3–5 mm, and 5–10 mm, as well as those aged for 0 h, 60 h, 120 h, and 240 h, were selected. The aging process was conducted in a laboratory oven (Model DHG-9140A, Shanghai Yiheng Scientific Instrument Co., Ltd., Shanghai, China) at a temperature of 135 °C. After aging, the mixtures were uniformly crushed using a laboratory crusher (Model FZ102, Shanghai Tiancheng Pharmaceutical Machinery Co., Ltd., Shanghai, China) to ensure consistent particle size. The prepared samples were then placed into 20 mL chromatography vials (Figure 2), resulting in a total of 20 samples. Each sample was tested three times using LF-NMR (Bruker Corporation, Billerica, MA, USA), and the average value was taken.

Determination of asphalt content was conducted using three different approaches to ensure accuracy and reliability. First, asphalt content was calculated in accordance with the specification JTG E20-2011 Test Methods of Asphalt and Asphalt Mixtures for Highway Engineering, using method T0705-2011. Second, the asphalt content was taken directly as the design value used during the mixture preparation, which was 5.3%. Third, the asphalt content was determined by applying the ignition method to the specimens after testing. The first method follows the T0705-2011 procedure specified in JTG E20-2011, which estimates asphalt content based on mass balance and material composition; while standardized and widely accepted, it is susceptible to experimental errors. The second method uses the designed asphalt content of 5.3%, offering a quick and convenient reference value but lacking the ability to reflect actual field conditions. The third method is the ignition method, which directly measures asphalt content by burning off the binder at high temperatures; it provides a realistic assessment of the asphalt remaining in the mixture after construction or testing, though it requires specialized equipment and may introduce minor inaccuracies due to potential aggregate degradation. Together, these methods offer a comprehensive evaluation framework for asphalt content determination. The detailed procedure for the ignition method is as follows:

The true asphalt content was determined using the combustion method in accordance with JJG (Traffic) 072-2006 Verification Regulation of Asphalt Content Tester (Combustion Method) [20]. Asphalt mixture samples were placed into the primary combustion chamber of an asphalt content tester (Model HTGQ-60L, Beijing Huatai Jingye Technology Co., Ltd., Beijing, China). To reach the specified experimental conditions, the temperature of the primary combustion chamber was raised from room temperature to 540 °C, and the secondary combustion chamber to 900 °C, within a maximum of 25 min. The asphalt content was then determined by calculating the mass difference of the asphalt mixture before and after combustion.

Since the mass difference after combustion does not solely represent the asphalt content, and due to the small sample size, which increases error, the basalt aggregates of 0–3 mm, 3–5 mm, and 5–10 mm were placed in the furnace to measure the loss on ignition. This was used to eliminate the error in the measured asphalt content due to aggregate loss. The corrected asphalt content is shown in Table 2.

For comparison, four asphalt samples with aging times of 0 min, 85 min, 175 min, and 340 min were prepared and subjected to low-field NMR testing. Different NMR parameters were used for asphalt and asphalt mixtures. The correlation between asphalt aging and asphalt mixture aging was examined through trends [2]. This study adopts a comparative approach based on approximate variation trends, wherein the normalized total peak area from the T_2_ spectra of asphalt mixture samples is compared with that of corresponding asphalt binder samples to validate the applicability of the proposed method.

To analyze the complex and multidimensional LF-NMR T_2_ spectral data, this study applied principal component analysis (PCA), an unsupervised machine learning technique commonly used for dimensionality reduction and feature extraction. Eight spectral parameters were selected as input variables, including start time, vertex time, end time, peak ratio, peak width, peak value, normalized peak area, and normalized total peak area. PCA was performed using SPSS Statistics 22 software to transform these correlated variables into a smaller set of uncorrelated principal components. This transformation allows for capturing the most significant variation in the original dataset while minimizing redundancy and noise. The resulting principal components were subsequently used as comprehensive indicators to characterize the aging behavior of asphalt mixtures. This machine learning approach facilitated an objective and data-driven interpretation of NMR spectral features in the context of asphalt aging evaluation.

## 3. Results and Discussions

### 3.1. Principal Component Analysis of Nuclear Magnetic Resonance Spectra

**Figure 3:**
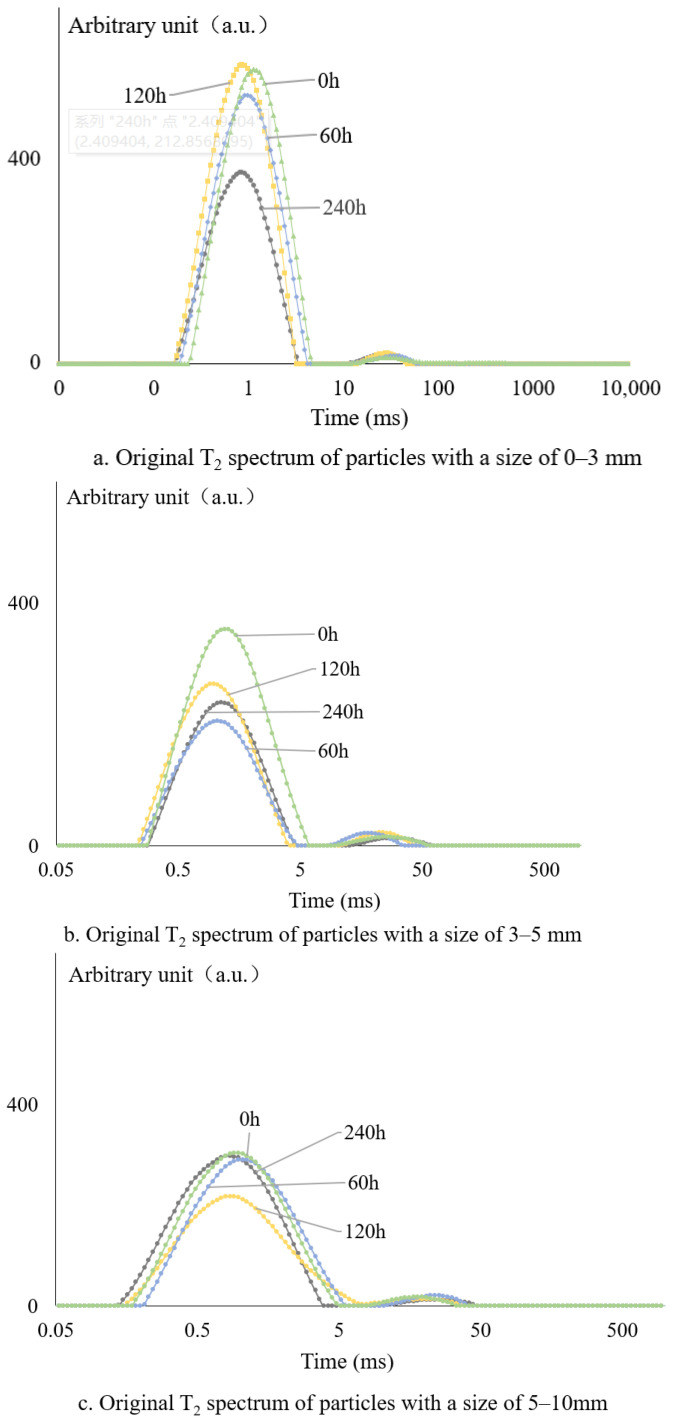

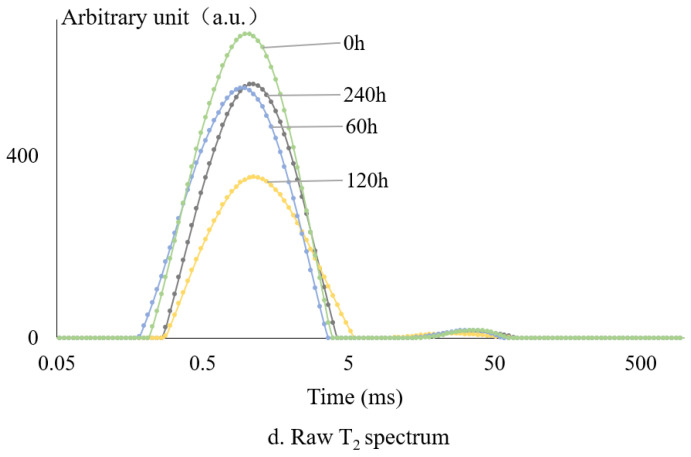
The original T2 spectrum of the mixtures at different aging times: (**a**) particle size 0–3 mm, (**b**) particle size 3–5 mm, (**c**) particle size 5–10 mm, and (**d**) mixtures.

Low-field nuclear magnetic resonance (LF-NMR) testing was conducted on asphalt mixtures with particle size ranges of 0–3 mm, 3–5 mm, and 5–10 mm, including unaged specimens and those subjected to long-term aging for 0, 60, 120, and 240 h, respectively. Long-term aged samples were selected to evaluate the influence of asphalt mass on LF-NMR response, based on the normalized asphalt content predicted by different methods. The NMR patterns of different particle sizes with aging time are as follows, see Figure 3.

The parameters in the T2 plot were introduced into SPSS for principal component analysis. The results are shown in Table 3. Generally, the components with principal component eigenvalues greater than or equal to 1 were selected as their principal components, so there existed three principal components: PCA1, PCA2, and PCA3, whose eigenvalues were ≥1. The variance contribution rate of the first two principal components was 84.36%, and the variance contribution rate of the first three principal components was 97.73%. Based on the first three principal components, most of the information of the original data can be summarized, and the principal component values of each sample were calculated based on the component score coefficient matrix, and the expressions of PCA1, PCA2, and PCA3 were calculated and outputted through SPSS software and expressed as follows:

PCA1 = −0.224X1 − 0.186X2 − 00.091X3 + 0.055X4 − 00.083X5 + 0.216X6 + 0.215X7 + 0.21X8 (4)

PCA2 = −0.007X1 + 0.019X2 + 0.353X3 − 00.284X4 + 0.359X5 + 0.061X6 + 0.149X7 + 0.166X8(5)

PCA3 = 0.296X1 + 0.549X2 + 0.110X3 + 0.574X4 + 0.099X5 + 0.351X6 + 0.198X7 + 0.177X8(6)

Equation:

X1—start time; X2—vertex time; X3—end time; X4—peak ratio; X5—peak width;

X6—peak value; X7—normalized peak area; X8—normalized peak total area.

**Table 3:** Relationship coefficient between principal components and indexes.

Component	Initial Eigenvalue		
	Total	Variance Proportion (%)	PCV (%)
1	4.17	52.15	52.15
2	2.58	32.22	84.36
3	1.07	13.37	97.73
4	0.11	1.35	99.09
5	0.05	0.57	99.66
6	0.02	0.28	99.93
7	0.01	0.07	100.00
8	0.00	0.00	100.00

The coefficients corresponding to each indicator in the two principal components (the weight coefficients of the principal components, see Table 4) can be obtained by dividing the data in Table 5 by the square root of the corresponding eigenvalues of the principal components. The larger the absolute value of the weight coefficients of the principal components, the greater the role of the corresponding indicators on the principal components. From Table 3, the first principal component is the most important with the largest variance contribution of 52.15%. In terms of the size of the absolute value of the weight coefficient, the ones that play a greater role in the 1st principal component are peak, normalized peak area, and normalized peak total area, which affect each other with the same change in size.

In summary, the NMR spectral parameter, i.e., the total area of the normalized peaks, for evaluating the aging of asphalt mixtures was deduced using Equation.

### 3.2. NMR Spectra of Different Particle Sizes and Aged Asphalt Mixtures

#### 3.2.1. The Raw T2 Spectrum of the Asphalt Mixture

**Figure 4:**
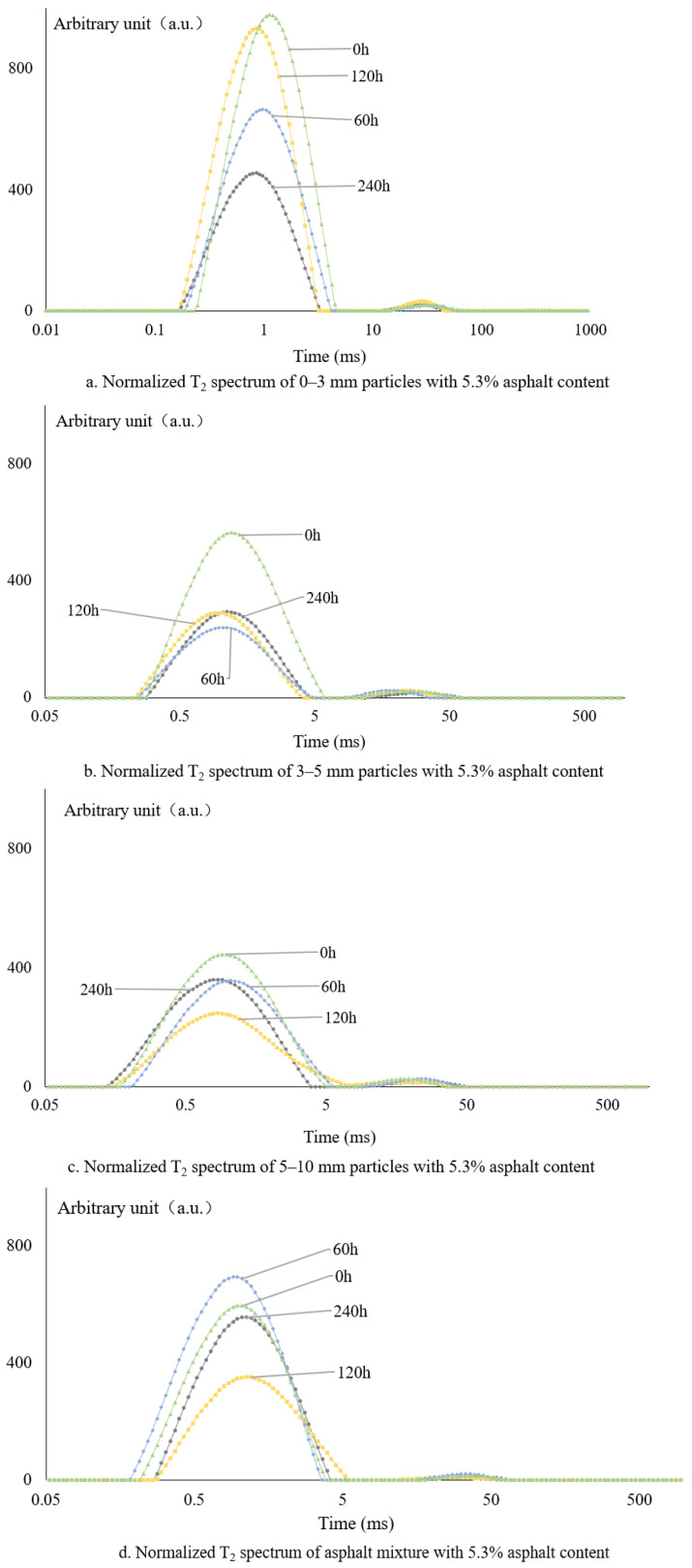
Normalized T2 spectrum of 5.3% designed asphalt content: (**a**) particle size 0–3 mm, (**b**) particle size 3–5 mm, (**c**) particle size 5–10 mm, and (**d**) bulk material raw T2.

Figure 4 shows that the original T2 spectrum of the asphalt mixture varies due to its different particle sizes. This rule is the same for different aging asphalt NMR spectra. The main reason is that the asphalt content of different-sized particles changes greatly. Generally speaking, the particle size is large, the unit weight of asphalt aggregate is small surface area, in the aggregate surface area of asphalt film thickness, its asphalt content is small, and vice versa.

When considering the normalization of asphalt content, the normalization calculation results of asphalt content will produce different results. Therefore, how to choose and determine the asphalt content is very important. If the design of the asphalt mixture is the optimal asphalt content, then the normalization results have different effects on the aggregate particle size of different asphalt mixtures. Another method adopts the calculation method, which is to calculate the asphalt content of the asphalt mixture with different particle sizes according to the specification. The third method is to use the combustion method to measure the asphalt content of the asphalt mixture with different particle sizes. The following are the results of the asphalt content using different methods.

#### 3.2.2. Normalized NMR Spectra Based on the Designed Asphalt Content

The asphalt content of all samples was estimated at 5.3%, and the total area of the normalized peak is shown in Table 6.

The normalized T2 profiles from 5.3% asphalt content are shown in Figure 4. According to Figure 4 and Table 6, the normalization of the NMR T2 map by the design of asphalt is basically similar to the original T2 map; that is, the asphalt aging T2 map is affected by the particle size of asphalt aggregate. However, the actual mixing content of fine particle asphalt is higher than the designed asphalt content, so the normalized area result is smaller than its value, and vice versa.

#### 3.2.3. Normalized NMR Spectra Based on Calculating the Asphalt Content

The average particle sizes of the three aggregate fractions, determined based on modulus-weighted calculations, were 9.5 mm, 4.75 mm, and 1.18 mm, respectively. The specific surface area (SA) of the aggregates was calculated using the surface-dry method formula specified in Chinese standards. Asphalt content was subsequently estimated based on the target asphalt film thickness and the relative specific surface area contributions of each particle size fraction, as shown in Table 7.

The table presents asphalt contents of 4.7%, 0.283%, and 0.383% for the 0–3 mm, 3–5 mm, and 5–10 mm particle size fractions, respectively. These values, derived through empirical calculations, exhibit substantial deviation from the design targets. Based on the asphalt content used during mixing, the normalized total peak area from LF-NMR measurements was calculated accordingly, as summarized in Table 8.

**Figure 5:**
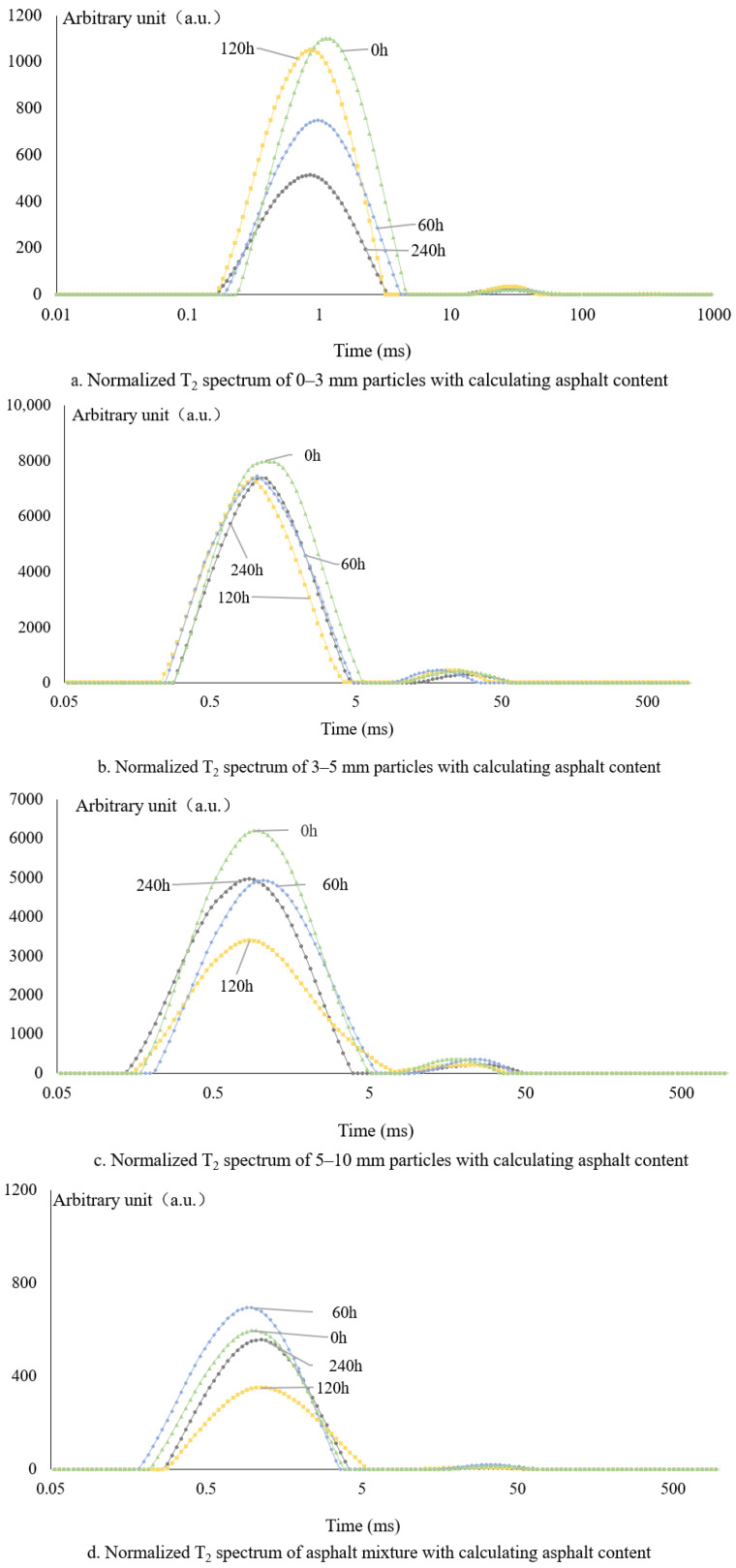
The normalized T2 spectrum using the asphalt content calculated: (**a**) particle size 0–3 mm, (**b**) particle size 3–5 mm, (**c**) particle size 5–10 mm, and (**d**) mixtures.

The asphalt content is calculated from Table 8 to obtain a normalized T2 figure as shown in Figure 5. Figure 5 illustrates the T_2_ nuclear magnetic resonance spectra normalized using the calculated asphalt contents for the three aggregate size fractions. Analysis of the T_2_ spectra reveals no consistent trend in signal variation with aging across different particle sizes. Regardless of the degree of aging, substantial discrepancies are observed in the behavior of the first and second peaks among the various size fractions. Both peak intensities and magnitudes suggest considerable error introduced during normalization. Specifically, the amplitude of the first peak, which indirectly reflects the normalized total peak area, ranges from 500 to 800 in lower cases to as high as 6000–7000 in others—a striking difference. This indicates that inaccuracies in asphalt content used for normalization can significantly distort both the quantitative parameters and the morphology of T_2_ spectra, deviating markedly from the original profiles and ultimately compromising the reliability of aging assessments. These findings suggest that the calculated asphalt content does not fully represent the actual binder distribution in the mixture.

#### 3.2.4. Normalized NMR Spectra Based on Asphalt Content by Combustion Method

**Figure 6:**
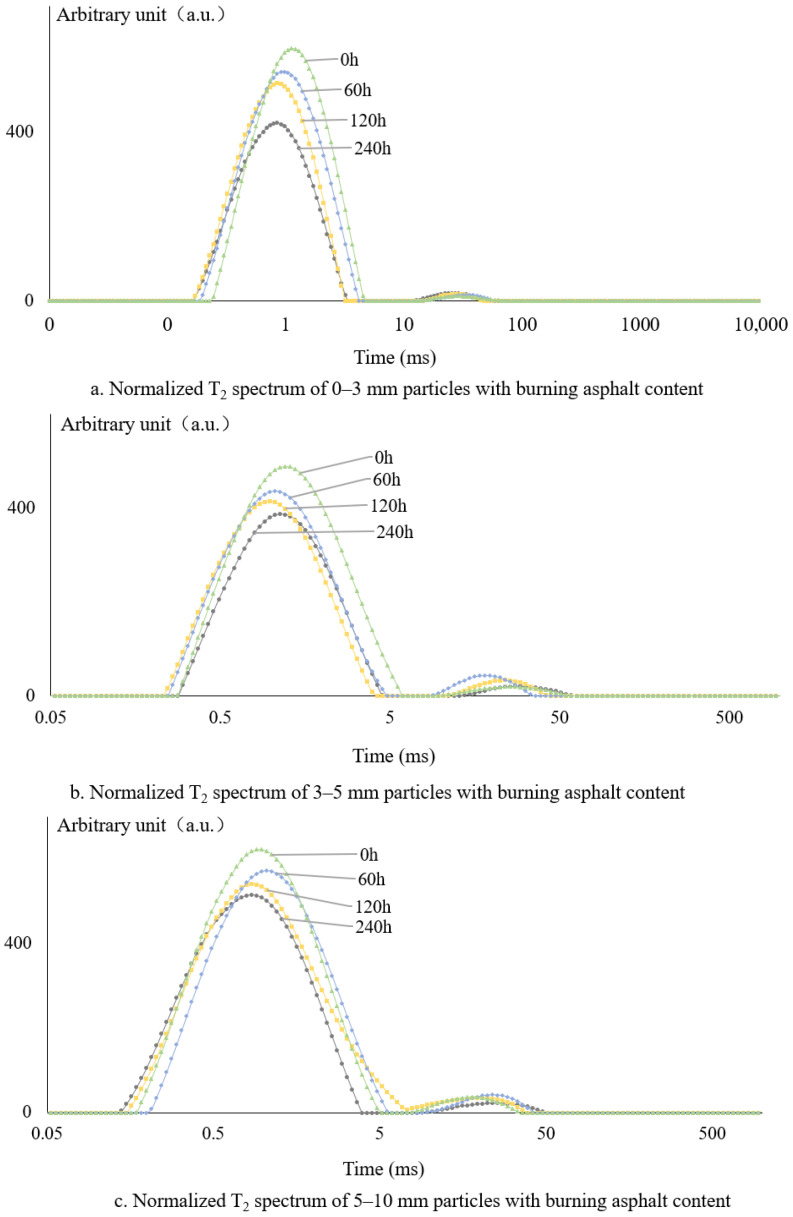

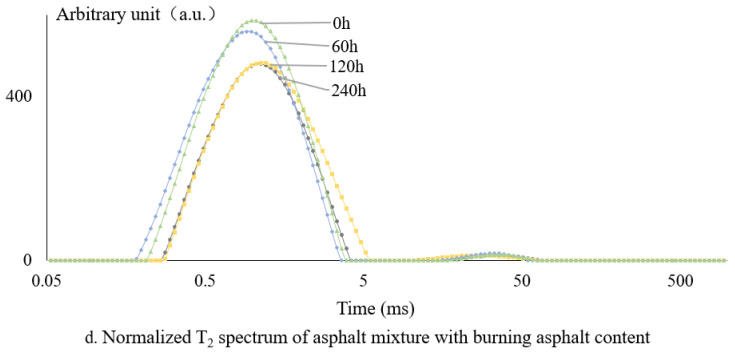
The normalized T2 spectrum of asphalt contents obtained by the combustion method: (**a**) particle size 0–3 mm, (**b**) particle size 3–5 mm, (**c**) particle size 5–10 mm, and (**d**) mixtures.

Results from previous studies indicate that the asphalt content predicted by the ignition method exhibits a discrepancy pattern similar to that observed between theoretical asphalt contents and the actual binder coating across different aggregate size fractions. The T2 map normalized by the true asphalt content obtained by the combustion method is shown in Figure 6.

**Figure 7:**
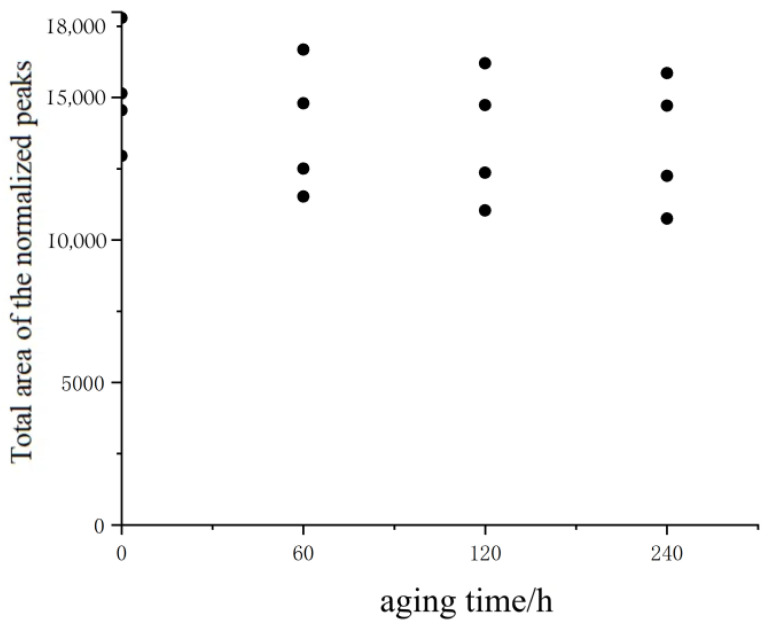
The total areas of the normalized peak with the actual asphalt contents.

The total area data of the normalized peaks of the actual asphalt content obtained from the combustion method are shown in Table 9 and Figure 7. The method of determining the asphalt content in the above three mixtures has a great influence on the asphalt content value. Undoubtedly, the size of the mixture aggregate has a great influence on its content, a fact proved by both the standard calculation method and the values obtained by the combustion method. The results of the asphalt content obtained by these three methods are therefore different. The actual content of the asphalt obtained by the calculation method and the mixture obtained by the combustion is also quite different.

From Table 9 and Figure 7, the trend line of aging over time is close. The total area of the normalized peaks decreased with the aging time of 0 h, 60 h, 120 h, and 240 h. The proportion of decline between adjacent aging sizes of 0–3 mm is 2.3%, 0.4%, and 0.2%, respectively, 11.0%, 4.3%, and 2.6% for 3–5 mm, 6.3%, 2.9%, and 2.1% for 5–10 mm, and 14.1%, 9.1%, and 7.8%, respectively. The difference before and after normalization mainly comes from the pitch content, and the huge size of the difference indicates that the pitch content has a great influence on the aging of the pitch mixture in the low-field NMR evaluation.

The asphalt content derived from both the formula calculation method and the design content method does not align with theoretical values, and the normalized data show no clear correlation with aging, making it unsuitable as a substitute for the ignition method for determining asphalt content. Therefore, for accurate asphalt content estimation, the ignition method remains the preferred approach in analyzing low-field nuclear magnetic resonance (LF-NMR) test results.

#### 3.2.5. Asphalt Aging-Normalized NMR Spectra

**Figure 8:**
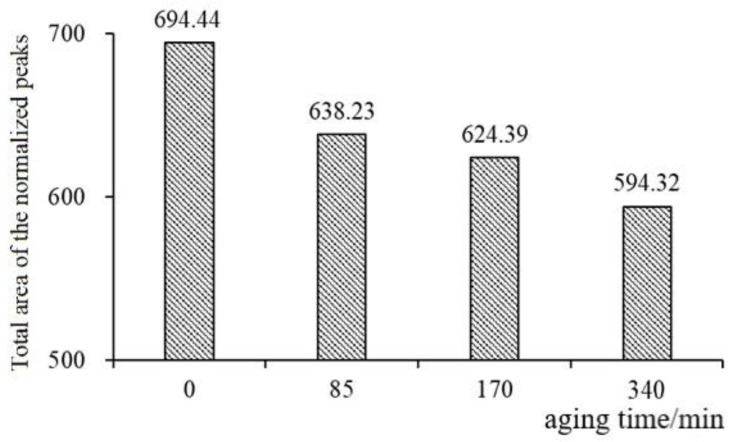
The total areas of the normalized peak with the actual asphalt contents.

Due to the use of different NMR acquisition parameters for asphalt binder and asphalt mixture, validation of LF-NMR as a reliable method for evaluating mixture aging was performed by testing asphalt samples subjected to four distinct aging levels. Trends observed in the binder’s T_2_ spectra were compared with those of the asphalt mixture to assess the consistency between asphalt aging and mixture aging behavior. Figure 8 shows the change pattern of the total area of the normalized peak of asphalt at different aging times. With the deepening of asphalt aging, the total area of normalized peak is decreasing, consistent with the trend of the asphalt mixture, and the change trend of the aging results of low-field NMR detection of asphalt mixture is proved.

## 4. Summary and Conclusions

The purpose of this study is to investigate the influence of the asphalt content on the NMR spectrum of the mixture in order to evaluate the aging degree of the asphalt. Low-field nuclear magnetic resonance (LF-NMR) was detected on the asphalt mixture with the same detection temperature, different particle sizes, and different aging degrees, and the main components of the NMR spectrum were found by a machine learning method and normalized. The asphalt content obtained by three different methods was studied and normalized, especially the law of normalization with aging. The conclusion is drawn from the result analysis:

(1) The principal component analysis method in SPSS is used to analyze the parameters that have a great influence on the principal components. From the perspective of the absolute value of the weight coefficient, the peak, normalized peak area, and normalized peak total area affect the first principal component. These three indicators actually influence each other and change together. The total normalized peak area can be used for evaluating the parameters of asphalt mixture aging by low-field NMR.
(2) Due to the different particle size, the asphalt content of the asphalt mixture of the same weight is different. This result is proved by both the calculation method and the combustion method, that is, the smaller the particle size of the sample, the greater the asphalt content. The asphalt content of the asphalt mixture is close to and less than the asphalt content during mixing. The asphalt content obtained by the three methods used in this study strongly influenced the normalization of the NMR spectra.
(3) As the aging time increases from 0 h to 60 h, 120 h and 240 h, the three-graded particles of asphalt mixture (0–3 mm, 3–5 mm, 5–10 mm) and the NMR spectrum approach the total area of the normalized peak, which are clearly related to the asphalt content.

To validate and contextualize the present findings, a comparative analysis with previous literature was conducted. Similar to earlier studies [5,10,11,12,13,17,19], the aging of asphalt mixtures led to a notable shift in the T_2_ relaxation peaks toward shorter relaxation times, reflecting increased molecular rigidity and reduced proton mobility. However, this study further demonstrates that the magnitude and pattern of these spectral shifts are significantly influenced by the asphalt content, which has not been sufficiently emphasized in prior work. In particular, higher asphalt content mitigated the extent of aging-induced T_2_ peak reduction, suggesting a buffering effect attributable to the increased binder phase. While the overall trends are consistent with previous reports, a few discrepancies were observed—primarily in the relative prominence of certain peaks—which can likely be attributed to differences in raw materials, sample preparation methods, and aging protocols. These variations highlight the necessity of considering mixture composition when interpreting NMR spectra in asphalt aging studies.

These findings indicate that the combination of LF-NMR and machine learning provides a promising non-destructive approach for road engineers to assess asphalt aging in situ. While the observed trends in spectral responses are generally applicable across mixtures, the method may require calibration when applied to different asphalt binders or aggregate gradations. Future studies will aim to extend this approach to modified asphalts and field-aged specimens, further enhancing its practical relevance and reliability in diverse engineering contexts.

## Figures and Tables

**Table 1:** Properties of 70 # base asphalt.

Properties	Measured Values	Specification
Penetration (25 °C, 0.1 mm)	69.1	60~80
Softening point (°C)	46.6	≥46
Ductility (15 °C, cm)	107	≥100

**Table 2:** The real content of asphalt after combustion correction (%).

Particle Sizes (mm)	0–3	3–5	5–10	Asphalt Mixtures
Unaged	6.6	5.1	4.0	5.2
Long-term aging 0 h	7.3	4.0	3.8	5.4
Long-term aging 60 h	8.1	4.3	2.6	5.0
Long-term aging 120 h	8.1	4.2	3.0	5.1
Long-term aging 240 h	8.7	4.0	3.3	5.2

**Table 4:** Principal component and index relationship coefficient.

Index	PCA1	PCA2	PCA3
X_1_ Starting time (ms)	−0.189	−0.007	0.296
X_2_ Vertex time (ms)	−0.186	0.019	0.549
X_3_ End time (ms)	−0.091	0.353	0.110
X_4_ Peak ratio (%)	0.055	−0.284	0.574
X_5_ Peak width (ms)	−0.083	0.359	0.099
X_6_ Peak	0.216	0.061	0.351
X_7_ Normalized peak area	0.215	0.149	0.198
X_8_ Normalized total peak area	0.210	0.166	0.177

**Table 5:** Component matrix.

Index	PCA1	PCA2	PCA3
X_1_ Starting time (ms)	−0.783	−0.018	0.317
X_2_ Vertex time (ms)	−0.778	0.048	0.588
X_3_ End time (ms)	−0.380	0.911	0.118
X_4_ Peak ratio (%)	0.231	−0.731	0.614
X_5_ Peak width (ms)	−0.346	0.926	0.106
X_6_ Peak	0.900	0.157	0.375
X_7_ Normalized peak area	0.895	0.383	0.212
X_8_ Normalized total peak area	0.874	0.427	0.189

**Table 6:** 5.3% asphalt mass normalized total peak area.

Particle Size/mm	Aging Time/h	Asphalt Weight/g	Unnormalized Total Peak Area	Total Normalized Peak Area
0–3	0	0.825	17,230	20,892
	60	0.629	14,277	22,707
	120	0.792	17,754	22,405
	240	0.591	14,245	24,121
3–5	0	0.632	6114	9671
	60	0.855	7946	9293
	120	0.918	8063	8780
	240	0.803	6553	8163
5–10	0	0.683	8694	12,724
	60	0.815	6716	8236
	120	0.883	8227	9319
	240	0.826	8159	9883
Asphalt mixtures	0	1.118	16,543	14,802
	60	0.790	1903	2410
	120	1.212	14,314	11,812
	240	0.742	8928	12,025

**Table 7:** Asphalt content for asphalt mixtures with different asphalt particle sizes.

Ore particle size/mm	9.5	4.75	2.36	1.18	0.6	0.3	0.15	0.075
Proportion of aggregates P_n_ (%)	0.9	26	38.6	8.5	7.5	6.6	3.1	2.1
Synthetic relative density of aggregates γ_sb_ (g/cm^3^)	2.833							
Asphalt content (%) P_b_	5.3							
Theoretical maximum relative density γ_t_ (g/cm^3^)	2.611							
Density of asphalt at 25 °C γ_b_ (g/cm^3^)	1.029							
Relative density of synthetic aggregates γ_se_ (g/cm^3^)	2.857							
The ratio of asphalt absorption by aggregates P_ba_ (%)	0.297							
Effective asphalt content in asphalt mixtures P_be_ (%)	5.018							
The mass passing percentage of aggregates P_i_ (%)	99.1	73.1	34.5	26	18.5	11.9	8.8	6.6
Surface coefficient Fai (m^2^/kg)	0.0041	0.0041	0.0082	0.016	0.029	0.061	0.123	0.3277
Aggregate specific surface area SA (m^2^/kg)	5.921							
Specific surface area ratio (%)	6.862	5.077	88.076					
Asphalt mass per kg aggregate (g)	3.843	2.834	49.323					
Different particle size asphalt content (%)	0.383	0.283	4.700					

**Table 8:** Calculated total area of the normalized peak of the asphalt content.

Particle Size/mm	Aging Time/h	Asphalt Quality/g	Unnormalized Total Peak Area	Total Normalized Peak Area
0–3	0	0.731	17,230	23,559
	60	0.558	14,277	25,606
	120	0.703	17,754	25,265
	240	0.524	14,245	27,201
3–5	0	0.034	6114	181,110
	60	0.046	7946	174,045
	120	0.049	8063	164,428
	240	0.043	6553	152,868
5–10	0	0.049	8694	176,087
	60	0.059	6716	113,976
	120	0.064	8227	128,957
	240	0.060	8159	136,766
Asphalt mixtures	0	1.118	16,543	14,801
	60	0.790	9313	11,791
	120	1.212	14,314	11,812
	240	0.742	8928	12,026

**Table 9:** Normalized peak area of asphalt content by combustion method.

Particle Size/mm	Aging Time/h	Asphalt Quality/g	Unnormalized Total Peak Area	Total Normalized Peak Area
0–3	0	1.138	17,230	15,144
	60	0.965	14,277	14,797
	120	1.205	17,754	14,737
	240	0.968	14,245	14,711
3–5	0	0.472	6114	12,952
	60	0.689	7946	11,529
	120	0.731	8063	11,037
	240	0.610	6553	10,748
5–10	0	0.489	8694	17,792
	60	0.403	6716	16,678
	120	0.508	8227	16,197
	240	0.515	8159	15,852
Asphalt mixtures	0	1.137	16,543	14,551
	60	0.745	9313	12,500
	120	1.158	14,314	12,363
	240	0.729	8928	12,245

## Data Availability

The original contributions presented in this study are included in the article. Further inquiries can be directed to the corresponding author.
